# RNA aptamer inhibitors of a restriction endonuclease

**DOI:** 10.1093/nar/gkv702

**Published:** 2015-07-15

**Authors:** Estefanía Mondragón, L. James Maher

**Affiliations:** 1 Neurobiology of Disease track, Mayo Graduate School, 200 First St SW, Rochester, MN 55905, USA; 2 Department of Biochemistry and Molecular Biology, Mayo Clinic College of Medicine, 200 First St SW, Rochester, MN 55905, USA

## Abstract

Restriction endonucleases (REases) recognize and cleave short palindromic DNA sequences, protecting bacterial cells against bacteriophage infection by attacking foreign DNA. We are interested in the potential of folded RNA to mimic DNA, a concept that might be applied to inhibition of DNA-binding proteins. As a model system, we sought RNA aptamers against the REases BamHI, PacI and KpnI using systematic evolution of ligands by exponential enrichment (SELEX). After 20 rounds of selection under different stringent conditions, we identified the 10 most enriched RNA aptamers for each REase. Aptamers were screened for binding and specificity, and assayed for REase inhibition. We obtained eight high-affinity (*K*_d_ ∼12-30 nM) selective competitive inhibitors (IC_50_ ∼20-150 nM) for KpnI. Predicted RNA secondary structures were confirmed by in-line attack assay and a 38-nt derivative of the best anti-KpnI aptamer was sufficient for inhibition. These competitive inhibitors presumably act as KpnI binding site analogs, but lack the primary consensus KpnI cleavage sequence and are not cleaved by KpnI, making their potential mode of DNA mimicry fascinating. Anti-REase RNA aptamers could have value in studies of REase mechanism and may give clues to a code for designing RNAs that competitively inhibit DNA binding proteins including transcription factors.

## INTRODUCTION

DNA binding proteins have a myriad of roles in the replication, transcription, repair and degradation of DNA. DNA restriction endonucleases (REases) are prokaryotic enzymes that cleave double-stranded DNA within or near their recognition sites. REases require divalent cations (typically Mg^2+^) for their activity and are thought to protect the host cell from the invasion of foreign DNA, such as viral DNA, by detecting DNA methylation status (1). It has been argued that REases arose as part of poison-antidote systems (2). Type II REases have been widely studied since their discovery in the 1970s (3,4) and have become essential tools for molecular cloning.

Our long-term aim is to design short RNA aptamers as specific inhibitors of DNA binding proteins such as transcription factors. We are approaching this challenge by first studying RNA aptamers selected from random libraries for DNA mimicry using various kinds of DNA binding protein targets. The premise that RNA aptamers might competitively inhibit DNA binding proteins is motivated by natural examples such as *Xenopus laevis* transcription factor IIIA (TFIIIA). This zinc finger protein engages the internal control region of the 5S RNA gene, as well as the 5S RNA transcript itself (5–9). The alternative binding states are mutually exclusive, such that the RNA functions as a transcription factor decoy to effect product inhibition. Other natural examples may exist, such as the transcription factor bicoid (9–12) and several other RNA decoys for DNA binding proteins have been artificially selected using SELEX (13,14) against forms of NF-κB (15–17), heat shock transcription factor (18), TFIIB (19) and RUNX1 (20). In the case of anti-NF-κB p50, the RNA aptamer has been studied at high resolution both free and bound to its protein target and has been shown to adopt a pre-formed tertiary structure that resembles closely the structure of DNA (21–23). Likewise, RUNX1 structure has been determined in DNA and RNA aptamer complexes. RNA is again observed to mimic DNA (24,25).

With the goal of collecting additional examples of RNA mimics of double-stranded DNA we applied SELEX to three Type II REases: BamHI, KpnI and PacI. We report the identification of several high-affinity RNA aptamers that act as selective competitive inhibitors of KpnI. These novel aptamers provide new examples for future structural analysis with the goal of decoy design principles.

## MATERIALS AND METHODS

### RNA libraries

**Figure 1.**
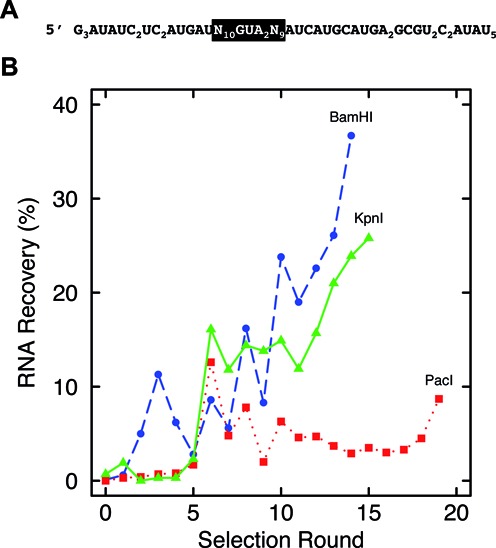
*Invitro* selection process. (**A**) RNA aptamer library format, random region and tetraloop highlighted in black. (**B**) Fraction of RNA recovered from selections against BamHI (blue circles), KpnI (green triangles) and PacI (red squares), as a function of selection round.

The RNA library used for the *in* *vitro* selections against REases were based on the *in* *vitro* and *in* *vivo* selections previously performed in our laboratory against transcription factor NF-κB (16,17). The results of these studies using a 60-nt random library showed that the minimal active domain was an imperfect 31-nt hairpin. This hairpin, defined by boundary and mutagenesis studies, has a 7-nt loop flanked by 17 critical nucleotides that are recognized by NF-κB. Far from being an asymmetric internal loop as predicted by secondary structure prediction algorithms, these nucleotides participate in a continuous stem composed of canonical and non-canonical interactions that result in striking mimicry of the major groove of a DNA double helix (17). Subsequent *in* *vivo* studies and re-selections were performed to select variants with improved activity in yeast (16). The resulting optimized aptamers displayed the same hairpin stem sequence but with a GUAA tetraloop replacing the original 7-nt loop. This interpretation was confirmed by subsequent high-resolution structural studies (21,22). We used this ‘scaffold’ as the basis for the present selections, reasoning that desired aptamers will mimic the structure of double-stranded DNA through variation of the theme exemplified by the anti-NF-κB aptamer. Therefore, the structured RNA libraries (Figure 1A) used for selection were 67-nt in length with a potential tetraloop (GUAA) flanked by two random regions: 10 nt upstream and 9 nt downstream of the tetraloop. These regions were then flanked by short complementary sequences supporting the weak stem, flanked by constant regions used for manipulation of the libraries (T7 RNA polymerase promoter, reverse transcriptase priming and polymerase chain reaction (PCR) priming sequences). Thus, the structure of the α-p50 aptamer provided inspiration for design of asymmetric random regions in the context of a small hairpin.

DNA oligonucleotides were synthesized commercially (IDT) and purified by the manufacturer using gel filtration chromatography. The synthetic DNA template for the selection pool was 5′-TA_2_TACGACTCACTATAG_3_ATATC_2_TC_2_ATGATN_10_G-TA_2_N_9_ATCATGCATGA_2_GCGT_2_C_2_ATAT_5_ where N indicates any base. Nucleotides in the random region were synthesized from a mixture of phosphoramidites adjusted for the relative coupling efficiency of each monomer. The library template was amplified by PCR using primers LJM-4485 5′-TA_2_TACGACTCACTATAG_3_ATATC_2_ and LJM-4486 5′A_5_TATG_2_A_2_CGCT_2_CATGCAT. Radiolabeled RNA aptamers were prepared by *in* *vitro* transcription of double-stranded DNA templates using the Ampliscribe T7 High Yield Transcription Kit (Epicentre) as described by the manufacturer, where standard 10-μl reactions included 2 μl [α-^32^P]-CTP (800Ci/mmol, 10 mCi/ml).

### Protein expression and purification

Commercial REases and purified preparations of BamHI (E111A mutant), KpnI and PacI for *in* *vitro* selection and binding assays were obtained from New England Biolabs.

### SELEX protocol

Naïve libraries for selection were prepared by PCR amplification of 5 pmol double-stranded DNA template. Because each template contains 19 random positions, the theoretical complexity of this library is 3 × 10^11^ unique sequences. A 5 pmol sample contains about 10 copies of each unique sequence. PCR reactions (100 μl) contained 20 mM Tris pH 8.4, 50 mM KCl, 4 mM MgCl_2_, 200 nM dNTPs, 400 nM of each primer, 20 nM DNA template and 0.1 U/μl recombinant Taq polymerase (Invitrogen). The optimum number of PCR cycles was determined by qualitative appearance of the amplification product on an 8% polyacrylamide gel (29:1 acrylamide:bisacrylamide), electrophoresed in 0.5× Tris-borate EDTA (TBE) buffer and stained with ethidium bromide. PCR was achieved by 2 min at 95°C as initial denaturation, followed by 3 cycles of 30 s at 95°C for denaturation, 30 s at 52°C as annealing, 30 s at 72°C for extension, followed by 5 cycles of 30 s at 95°C for denaturation, 30 s at 65°C as annealing, 30 s at 72°C for extension and final extension for 5 min at 72°C. Products were separated by electrophoresis through 2.5% agarose gels in 1× TAE buffer and the major product purified using a QIAquick gel extraction kit (Qiagen). Initial RNA pools were transcribed from 6 pmol of DNA template (representing ∼10^12^ sequences) using an Ampliscribe T7 Transcription Kit (Epicentre) as described above. RNA was purified by denaturing 10% polyacrylamide gel electrophoresis (29:1 acrylamide:bisacrylamide, 8 M urea, 0.5X TBE), visualized by UV shadowing, excised and eluted from gel pieces overnight at room temperature with agitation in 2× PK buffer (200 mM Tris pH 7.6, 2.5 mM ethylenediaminetetraacetic acid (EDTA), 300 mM NaCl and 2% sodium dodecyl sulphate). The eluted material was extracted with an equal volume of phenol/chloroform (1:1, v/v) and RNA was precipitated by the addition of 2.5 volumes of ethanol. For initial selections, 23 pmol of pool RNA representing ∼43 copies each of 3 × 10^11^ DNA templates was used as a 0.46 nM solution in a volume of 50 ml in the presence of 2 nM target protein in the appropriate selection buffer (SB) such that REases are not catalytically active: BamHI(E111A): 10 mM HEPES pH 7.5, 400 mM NaCl, 1 mM MgCl_2_; KpnI: 10 mM HEPES pH 7.5, 100 mM NaCl, 1 mM CaCl_2_; and PacI: 10 mM HEPES pH 7.5, 10 mM NaCl, 1 mM CaCl_2._ Prior to selection all RNA pools were incubated in SB at 70°C for 5 min and allowed to cool to room temperature to promote RNA folding. Selections were performed by incubation of RNAs for 45 min in SB buffer with or without protein followed by vacuum partitioning using nitrocellulose filtration (Millipore HAWP02500, 0.45 μm HA filters). Unbound RNAs were washed from the filter using two aliquots of 10 ml SB. Captured RNA was then quantitated by Cerenkov radiation detected on filters using the ^3^H setting of a scintillation counter. Bound RNA was eluted in 500 μl 2× PK buffer at 65°C for 30 min. The eluted solution was extracted with an equal volume of phenol:chloroform (1:1) and RNA precipitated from ethanol using 40 μg of glycogen as carrier. Before round 3 and subsequent odd rounds negative selections were performed by exposing the RNA library to nitrocellulose filter strips and processing the supernatant for selection. To prepare cDNA, one-third of the recovered RNA was reverse transcribed using primer LJM-4486 (5′-A_5_TATG_2_A_2_CGCT_2_CATGCAT). Prior to the addition of MMLV reverse transcriptase and dNTPs, the primer was annealed by incubating for 5 min at 65°C and 5 min at room temperature. Each 40-μl reverse transcription reaction contained 1.25 μM primer LJM-4486, 400 μM dNTPs, 50 mM KCl, 6 mM MgCl_2_, 50 mM Tris pH 8.0 and 2 μl 200 U/μl MMLV reverse transcriptase (Invitrogen.) Reactions were incubated at room temperature for 10 min and at 37°C for 30 min. One-half of the each reaction was used for optimization PCR. After PCR amplification of the remaining cDNA, DNA libraries were purified using a PCR Purification Kit (Qiagen). To prepare RNA pools for subsequent rounds of selection, 200 ng (4 pmol) purified DNA was transcribed as previously described. To monitor binding and recovery of radiolabeled RNAs at each round of selection, nitrocellulose filter radioactivity was compared to a fraction of total radioactivity to determine the fraction of RNA bound to the protein target. Twenty rounds of selection were performed for each enzyme with maximum RNA recovery for BamHI reached at cycle 14, for KpnI at cycle 15 and for PacI at cycle 19 (Figure 1B).

### Cloning

The double-stranded DNA libraries of rounds 14, 16 and 18 for BamHI (E111A), 15, 16 and 18 for KpnI, and 18, 19 and 20 for PacI were ligated into the pGEM-TEasy vector using a TA cloning kit (Promega). Clones with coherent sequences were aligned using multiple sequence alignment tool MUSCLE (EMBL).

### Qualitative binding screen

RNAs were 3′ end-labeled by ligation of [^32^P]-cytidine 3′,5′ bis(phosphate), (3000 Ci/mmol; 10 mCi/ml), using T4 RNA ligase (New England Biolabs). Unincorporated nucleotides where removed by size exclusion chromatography using Chromaspin TE-10 gel filtration columns (Clontech). Binding reactions (25 μl) contained the appropriate SB, 4 nM RNA and 20 nM target protein. Reactions were incubated for 30 min at 37°C. Binding reactions were supplemented with glycerol loading dye and analyzed by electrophoresis through 8% polyacrylamide gels in 1× TBE at 3W for 1 h. Gels where then dried and exposed overnight using storage phosphor imaging technology (Typhoon FLA-7000).

### Quantitative binding assay

RNA aptamer binding to target REases was determined by electrophoretic gel mobility shift assay. RNAs were transcribed from the appropriate double-stranded DNA template and radiolabeled as described above. Binding reactions contain radiolabeled aptamer (13 nM) and the indicated concentration of KpnI in New England Biolabs 1.1 buffer (10 mM Bis-Tris-Propane-HCl, pH 7, 10 mM MgCl_2_, 100 μg/ml bovine serum albumin (BSA)) for 30 min at 37°C. Samples where then directly loaded onto 8% polyacrylamide gels (19:1 acrylamide:bisacrylamide) and electrophoresed in 1× TBE buffer. Gels were dried for 30 min and signal detected by storage phosphor imaging. Image quantitation involved ImageJ software (NIH) with the equilibrium dissociation constant estimated by least squares fitting to a modified Hill equation using R software (version 2.13.0):

(1) \begin{equation*} \theta = \theta _{{\rm min}} + \left[ {\frac{{\theta _{{\rm max}} - \theta _{{\rm min}} }}{{1 + \left( {\frac{{K_{\rm d} }}{{P_{\rm t} }}} \right)^h }}} \right] \end{equation*}

where *θ* is the fraction of RNA bound, *K*_d_ is the dissociation constant, *P*_t_ is the total protein concentration and *h* is the Hill coefficient.

### Restriction inhibition assay

**Figure 2.**
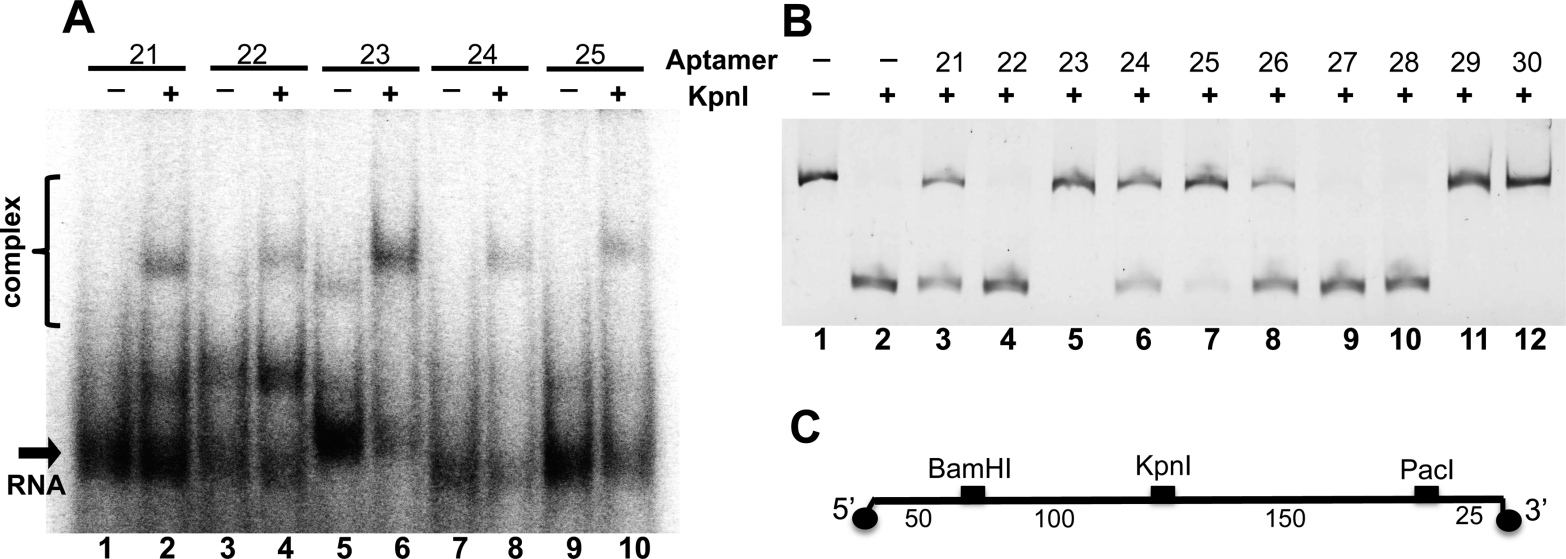
Example gels showing results of qualitative screens for aptamer activity. (**A**) Electrophoretic gel mobility shift binding screen. Radiolabeled aptamer candidates (4 nM) were exposed to their corresponding protein targets, in this case KpnI (20 nM; even lanes). (**B**) Restriction inhibition screen. Fluorescent DNA probe (20 nM, see panel **C**) was exposed to REase that had been incubated with a high concentration of the indicated RNA aptamers (40 μM). The examples shown are RNA aptamers against KpnI.

A fluorescent 321-bp double-stranded DNA probe was prepared by PCR from five overlapping oligonucleotides and two primers synthesized with 6-FAM fluorescein phosphoramidites. The probe contains unique recognition sites for BamHI, KpnI and PacI in an asymmetric pattern (Figure 2C). Qualitative inhibition assays included RNA aptamer (40 μM) in the digestion buffer recommended by the enzyme manufacturer (New England Biolabs): buffer 3.1 for BamHI, buffer 1.1 for KpnI and CutSmart buffer for PacI. Reaction buffer with RNA aptamers were heated to 70°C for 5 min then allowed to cool to room temperature. The appropriate commercial REase was then added to a final concentration of 0.2 U/μl as indicated and reactions incubated for 30 min at 37°C. Fluorescent 321-bp target probe (20 nM) was added and digestion allowed to proceed for 30 min at 37°C. Reactions were terminated by heating for 15 min at 65°C. After addition of glycerol loading dye, samples were analyzed by electrophoresis through 8% polyacrylamide gels (29:1 acylamide:bisacylamide) in 0.5× TBE buffer. Reaction products were imaged using the Typhoon FLA-7000 instrument at a wavelength of 475 nm. When desired, assay quantitation was implemented by image analysis (Image J) and the RNA aptamer concentration necessary for 50% enzyme inhibition (IC_50_) estimated by curve fitting:

(2) \begin{equation*} f = \frac{1}{{1 + \left[ {\frac{{{\rm IC}_{50} }}{{[A]}}} \right]^S }} \end{equation*}

where *f* is the fraction of active enzyme, *S* is the slope factor and *A* is the aptamer concentration.

### Curve-fitting

A weighted non-linear least squares method was used to minimize the cost function *C*(**p**) with respect to the free parameters **p**

(3) \begin{equation*} \chi ^2 = \mathop {\min }\limits_{\rm p} \quad C({\bf p}) = \mathop {\min }\limits_{\rm p} \sum\limits_{m = 1}^M {\left( {\frac{{\bar \mu _m - f(c_m ;{\bf p})}}{{\sigma _m }}} \right)^2 } \end{equation*}

where *M* is the number of data points being fit, and (corresponding to the *m*-th data point) *c_m_* are concentrations, $\bar \mu _m$ are experimentally determined mean values, $\sigma _m$ are standard deviations of $\bar \mu _m$ and $f(c_m ;{\bf p})$ are theoretical predictions of the model (either Hill equation or 2-parameter logistic model).

### Monte Carlo estimation of uncertainty

Simulated datasets were generated from the experimental data by adding Gaussian noise, i.e. randomly sampling from the normal distributions $N_m (\mu = \bar \mu _m ,\sigma = \sigma _m )$, corresponding to the experimental mean and standard deviations of the *m*-th data point. These simulated data sets ($N_m^\varepsilon$ for $\varepsilon = 1, \ldots \varepsilon _{\max }$) were then minimized against the Hill equation (for *K*_d_) or 2-parameter logistic model (for IC_50_) to determine ${\bf p}_{\min }^\varepsilon$

(4) \begin{equation*} \mathop {\min }\limits_{{\rm p}^\varepsilon } \sum\limits_{m = 1}^M {\left( {\frac{{N_m^\varepsilon - f(c_m ;{\bf p}^\varepsilon )}}{{\sigma _m }}} \right)^2 } \end{equation*}

For a given parameter *p_k_*, the standard deviation was estimated as:

(5) \begin{equation*} \sigma _{p_k } = \sqrt {\frac{1}{{\varepsilon _{\max } - 1}}\sum\limits_{\varepsilon = 1}^{\varepsilon _{\max } } {\left( {p_{\min ,k}^\varepsilon - p_k } \right)^2 } } \end{equation*}

where *ε*_max_ was set at 10 000 and $\bar p_k$ (the mean value) is:

(6) \begin{equation*} \bar p_k = \frac{1}{{\varepsilon _{\max } }}\sum\limits_{\varepsilon = 1}^{\varepsilon _{\max } } {p_{\min ,k}^\varepsilon } \end{equation*}

Additionally, the relative bias in the parameter *p_k_* is given by,

(7) \begin{equation*} B_k = \frac{{\left| {p_{\min ,k} - \bar p_k } \right|}}{{p_{\min ,k} }} \end{equation*}

For all of the parameters, the median relative bias was 2.4%, giving confidence in the method. For *K*_d_ and IC_50_, the median relative biases were 0.5 and 3.8%, respectively.

### In-line probing

RNA aptamers were dephosphorylated and labeled at 5′ termini using γ- [^32^P]-ATP (6000 Ci/mmole; 10 mCi/ml), using calf intestinal alkaline phosphatase (CIP) and T4 polynucleotide kinase (New England Biolabs), followed by gel purification. All reactions contained 10^5^ radioactive counts per minute of radiolabeled aptamer RNA. In-line probing reactions (10 μl) contained 50 mM Tris–HCl (pH 8.3 at 20°C), 20 mM MgCl_2_ and 100 mM KCl. Reactions were incubated at room temperature for 24 or 48 h. RNase T1 treatment was performed in 25 mM sodium citrate pH 5.0, 0.1 U/μl RNase T1, in the presence of 5 M urea and 0.75 mM EDTA (pH 8.0 at 20°C). Reactions were incubated at 55°C for 3 min. Partial alkaline hydrolysis reactions were performed in 50 mM Na_2_CO_3_ pH 9.0, 1 mM EDTA, incubated at 95°C for 5 min. All reactions were terminated by ethanol precipitation. Vacuum-dried pellets were then resuspended in 8 μl deionized formamide gel-loading solution and immediately analyzed by gel electrophoresis through 10% denaturing polyacrylamide sequencing gels (10%, 29:1 acrylamide:bisacrylamide) in 1× TBE buffer. Electrophoresis was at 48 W for 3 h. Gels were then transferred to blotting paper, dried for 1 h and signal detected by storage phosphor imaging. Signal analysis was performed using SAFA (26). Extracted and quantitated band intensities were then normalized by subtracting background from unmodified sample and dividing by the partial hydrolysis signal, yielding values to generate a colored heat plot.

### Competition gel shift assay

Binding reactions were performed in digestion inhibition conditions (10 mM HEPES pH 7.0, 100 mM NaCl, 1 mM Ca^2+^ and 1 μg/ml BSA). Reaction buffer with RNA aptamers was heated to 70°C for 5 min and allowed to cool to room temperature. The appropriate commercial REase was then added to a final concentration of 4 U/μl and reactions incubated for 30 min at 37°C. Fluorescent DNA probe (20 nM) was then added with incubation for 30 min at 37°C. After addition of glycerol loading dye, samples were immediately analyzed by electrophoresis through 8% polyacrylamide gels (29:1 acylamide:bisacylamide) in 0.5× TBE buffer. Gels were imaged using the Typhoon FLA-7000 instrument at a wavelength of 475 nm.

### CD spectroscopy

RNA samples were transcribed *in* *vitro* from synthetic duplex DNA templates as previously described, gel purified and extracted from gels using an Elutrap electroelution system, buffer exchanged into 100 mM sodium cacodylate buffer, pH 6.6 and concentrated to ∼300 ng/μl. Circular dichroism spectra were collected using a JASCO J-810 spectropolarimeter. Wavelength scans from 200 to 320 nm were collected every 1 nm at a speed of 100 nm/min at a sensitivity of 100 mdeg with accumulation of 5 counts at 25°C using a 1 mm cuvette.

## RESULTS

### *In* *vitro* selection

SELEX against Type II REases BamHI, KpnI and PacI was performed using a 67-nt RNA library containing blocks of 10 and 9 random nucleotides flanking a 5′-GUAA tetraloop (Figure 1A) within a weak stem-loop structure modeled after the framework of the previously-studied anti-NF-κB p50 RNA aptamer [(12,17,23) and see ‘Materials and Methods’ section]. To suppress potential RNA aptamer cleavage by KpnI and PacI, selections were performed in the presence of Ca^2+^. Selections with the catalytically inactive BamHI (E111A) mutant were performed in Mg^2+^. Preliminary experiments revealed that each enzyme required different salt conditions to maintain selection stringency (fraction of recovered RNAs less than 1% in early rounds; data not shown). These individual conditions were then used for each enzyme throughout the 20 rounds of selection. A negative selection against nitrocellulose filter strips was performed after odd cycles to reduce aptamers enriched for binding to the selection matrix. After 20 rounds we studied aptamers from three different rounds of selection including the most enriched cycles for each REase. Anti-BamHI, anti-KpnI and anti-PacI RNA aptamers were retained at 36, 26 and 9% after 14, 15 and 19 selection cycles, respectively (Figure 1B). After cloning, complete sequences were obtained for ∼50 aptamers from each of the sequenced cycles and analyzed using sequence alignment tool MUSCLE (EMBL) (Table 1). The number of unique sequences in each library tended to decrease with more cycles, as expected. Interestingly, although selections against BamHI gave the highest fraction of RNA recovery (36% in cycle 14), 41 unique sequences were found in that pool and the aptamer with highest abundance was found only three times. In contrast, cycle 15 of KpnI selection gave only 9 unique sequences and the most abundant was found 19 times. Thus, libraries were not equally converged (Table 1). The basis for this difference is not known, but different protein characteristics and ionic conditions of selections presumably played roles in the effect.

**Table 1.** Dominant aptamer sequences for each of three target REases

Target	Round	Sequenced clones	Unique sequences	Abundance of most selected sequence	Random regions of dominant aptamers
					**NNNNNNNNNN**GUAA**NNNNNNNNN**
**BamHI**	14^a^	48	41	3	**AAUAAAUCGA**GUAA**UAAUCUAAU**
	16	50	37	5	**UAUAUAUCAA**GUAA**UAAUCAUAA**
	18	48	36	5	**AAAUCGUAUA**GUAA**UAAUUAGUA**
**KpnI**	15^a^	54	9	19	**GGCGAAAAGC**GUAA**CCGCGGCGA**
	16	52	8	15	**GGCGAAAAGC**GUAA**CCGCGGCGA**
	18	58	8	15	**GGCGAAAAGC**GUAA**CCGCGGCGA**
**PacI**	18	46	12	18	**AUAUAUAAUU**GUAA**AUUCUAAUG**
	19^a^	36	13	12	**AUAUAUAAUU**GUAA**AUUCUAAUG**
	20	54	16	13	**AUAUAUAAUU**GUAA**AUUCUAAUG**

^a^Library with highest RNA recovery.

### Binding and inhibition screens

The most enriched aptamers for each target enzyme were initially characterized by testing their ability to both bind and inhibit each REase target. Binding affinity and specificity were screened using a qualitative gel shift assay in which 4 nM RNA aptamer was incubated with or without five-fold molar excess REase and then analyzed by electrophoretic gel mobility shift assays in 8% polyacrylamide gels. Figure 2A shows anti-KpnI aptamers 21–25 as an example. There was evidence of target binding for all aptamers when exposed to cognate REases (Table 2). Aptamer binding was generally specific for the selection target, but a few aptamers (e.g. anti-KpnI aptamers 17 and 27) showed weak non-specific interaction with BamHI.

**Table 2.** Summary of RNA aptamer binding to protein target (4 nM) and enzyme inhibition at an aptamer concentration of 40 μM

Aptamer	Protein target	BamHI	KpnI	PacI	BamHI	KpnI	PacI
		*protein binding*^a^			*enzyme inhibition*^a^		
1/9/10	BamHI	+	−	−	−	−	−
2	BamHI	+	−	−	−	−	−
3/7	BamHI	+	−	−	−	−	−
4	BamHI	+	−	−	−	−	−
5/13	BamHI	+	−	−	−	−	−
6/12	BamHI	+	−	−	−	−	−
8	BamHI	+	−	−	−	−	−
15	KpnI	−	+	−	−	+	−
16/23/29	KpnI	−	+	−	−	+	−
17/22/28	KpnI	±	+	−	−	−	−
18/21/26	KpnI	−	+	−	−	±	−
19	KpnI	−	+	−	−	+	−
20	KpnI	−	+	−	−	+	−
24	KpnI	−	+	−	−	±	−
25	KpnI	−	+	−	−	+	−
27	KpnI	±	+	−	−	−	−
30	KpnI	−	+	−	−	+	−
38/33	PacI	±	−	±	−	+	−
39/34/36	PacI	−	−	±	−	−	−
40/51	PacI	−	−	±	−	−	−
41/32	PacI	−	−	±	−	−	−
44	PacI	−	−	±	−	−	−
45	PacI	−	−	±	−	−	−
46/50	PacI	−	−	+	−	−	−
52	anti-p50	−	−	−	−	−	−
31	T2	−	−	−	−	−	−

^a^Binding/inhibition, (+); partial binding/inhibition, (±); no binding/inhibition (-).

Initial qualitative REase inhibition screening was performed by testing the capacity of a single high concentration of each aptamer to inhibit BamHI, KpnI and PacI cleavage. A 321-bp fluorescent duplex DNA probe carrying single recognition sequences for each REase was designed for this purpose Figure 2C. Example data are shown in Figure 2B. To assess specificity, all aptamers were screened for their ability to inhibit all three of the target enzymes. Interestingly, only anti-KpnI RNA aptamers showed both selective binding and restriction inhibition (Table 2).

### Quantitating KpnI binding by RNA aptamers

**Figure 3.**
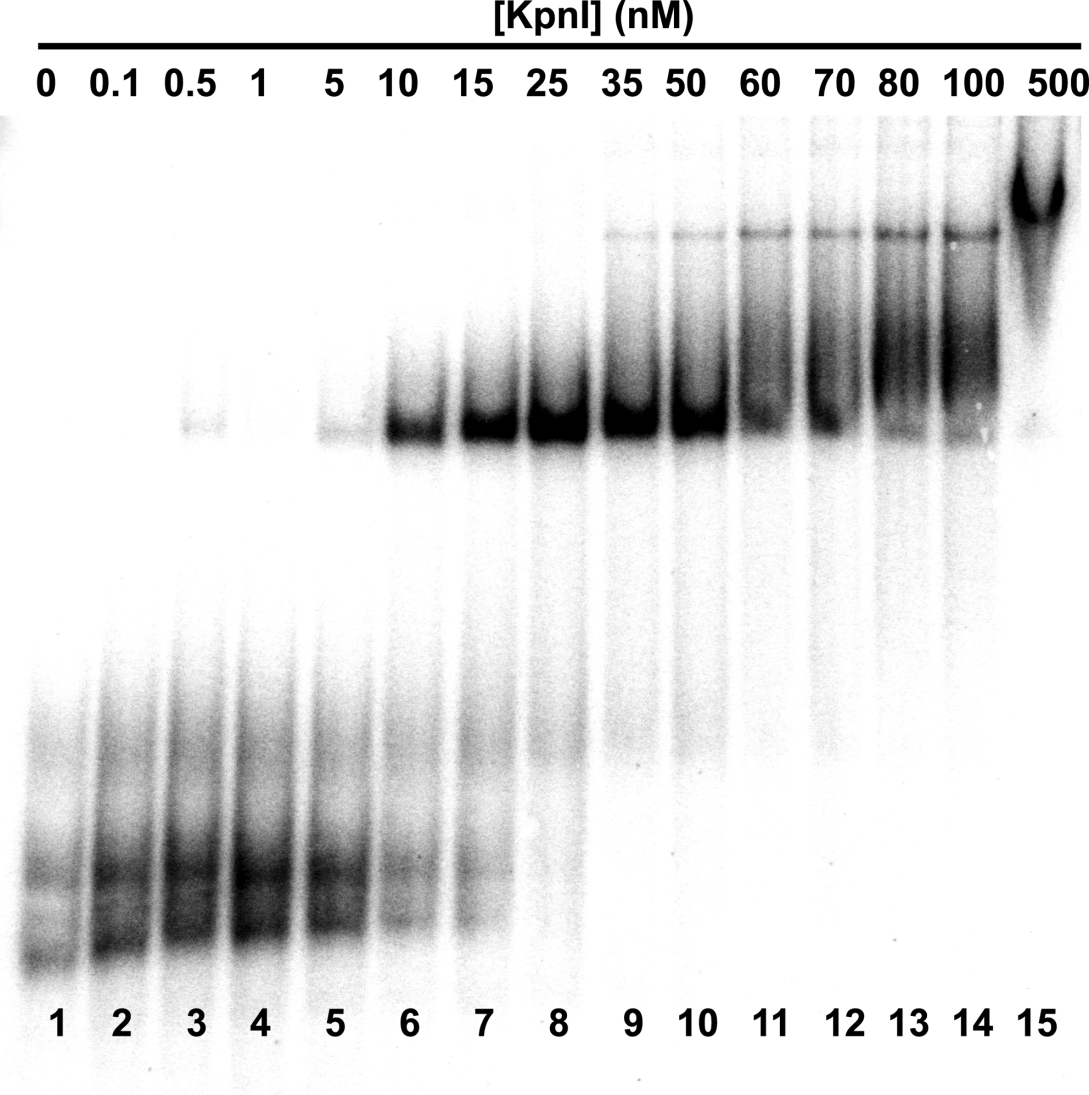
Example of anti-KpnI aptamer 20 binding to KpnI as measured by quantitative electrophoretic gel mobility shift assay. A total of 13 nM radiolabeled RNA aptamer titrated with increasing concentrations of KpnI as indicated.

**Figure 4.**
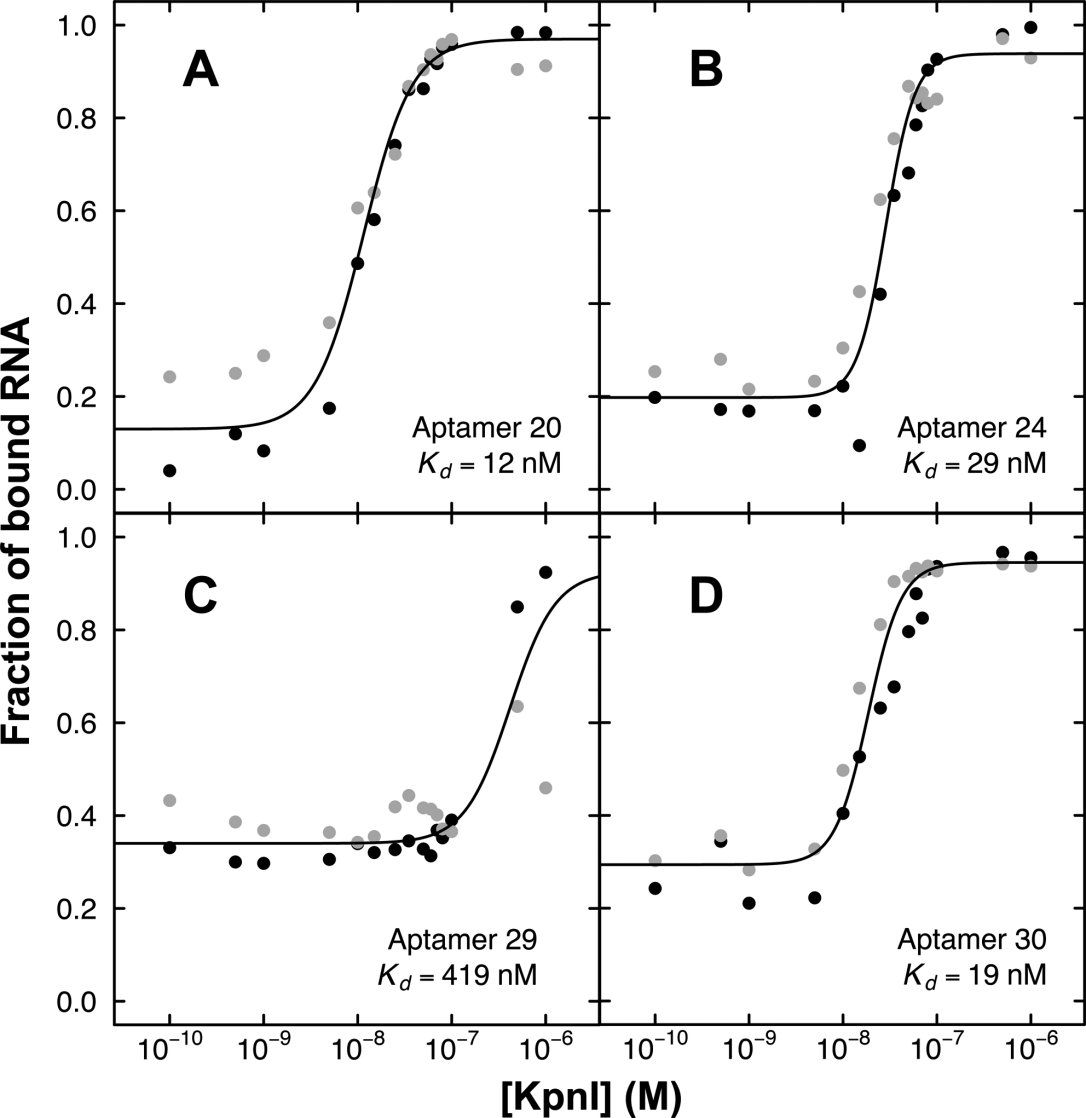
Quantitation of KpnI binding affinity by electrophoretic gel mobility shift assay for (**A**) aptamer 20; (**B**) aptamer 24; (**C**) aptamer 29; and (**D**) aptamer 30. Replicate data are shown as black and gray points.

To evaluate quantitatively, the affinities of four representative anti-KpnI aptamers, aptamer 20, 24, 29 and 30, (Table 3) we used an electrophoretic gel mobility shift assay where radiolabeled RNA aptamers (15 nM) were titrated with increasing concentrations of KpnI (aptamer 20 example shown in Figure 3). Gel images were quantitated and equilibrium dissociation constant (*K*_d_) and uncertainty values estimated by fitting as described in ‘Materials and Methods’ section. Summary data are shown in Figure 4 and Table 4.

**Table 3.** Sequences of representative anti-KpnI aptamers^a^ and aptamer 20 mutants

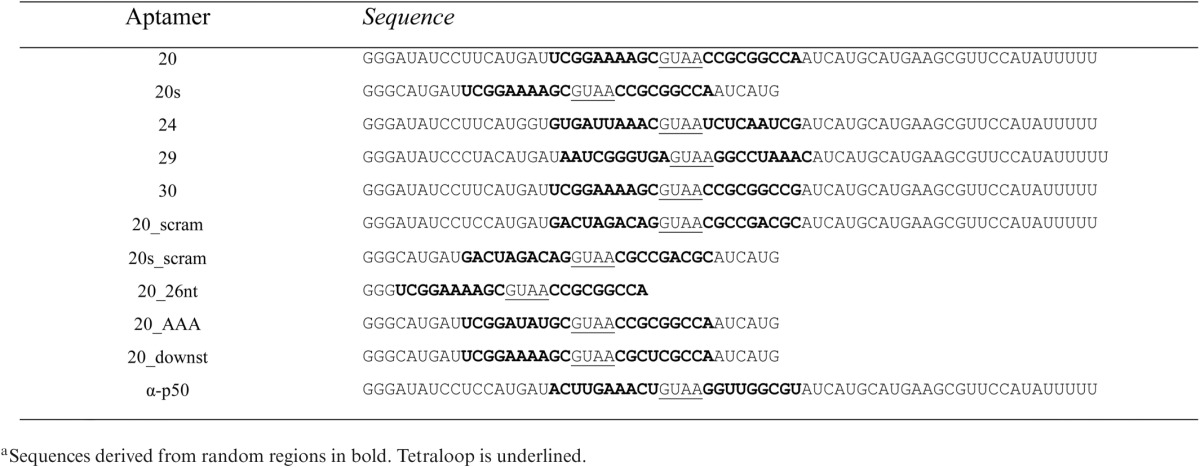

**Table 4.** Apparent equilibrium dissociation constant (*K*_d_), Hill coefficient (*h*), maximum fraction of bound RNA (*f*_max_) and minimum fraction of bound RNA (*f*_min_) for selected aptamers binding to KpnI

Aptamer^a^	*K_d_*	*h*	*f*_max_	*f*_min_
20	11.6 ± 2.3 nM	1.68 ± 0.48	0.97 ± 0.03	0.13 ± 0.06
24	28.7 ± 5.2 nM	2.83 ± 0.95	0.94 ± 0.04	0.20 ± 0.02
29	419 ± 46.6 nM	1.17 ± 1.18	1.00 ±0.07	0.42 ±0.13
30	18.6 ± 4.5 nM	2.45 ± 0.85	0.95 ± 0.02	0.29 ± 0.02

^a^Parameters reported reflect the mean and standard deviation of 10 000 fits to experimental data (at least two replicates) with added Gaussian noise (see ‘Materials and Methods’ section).

**Figure 5.**
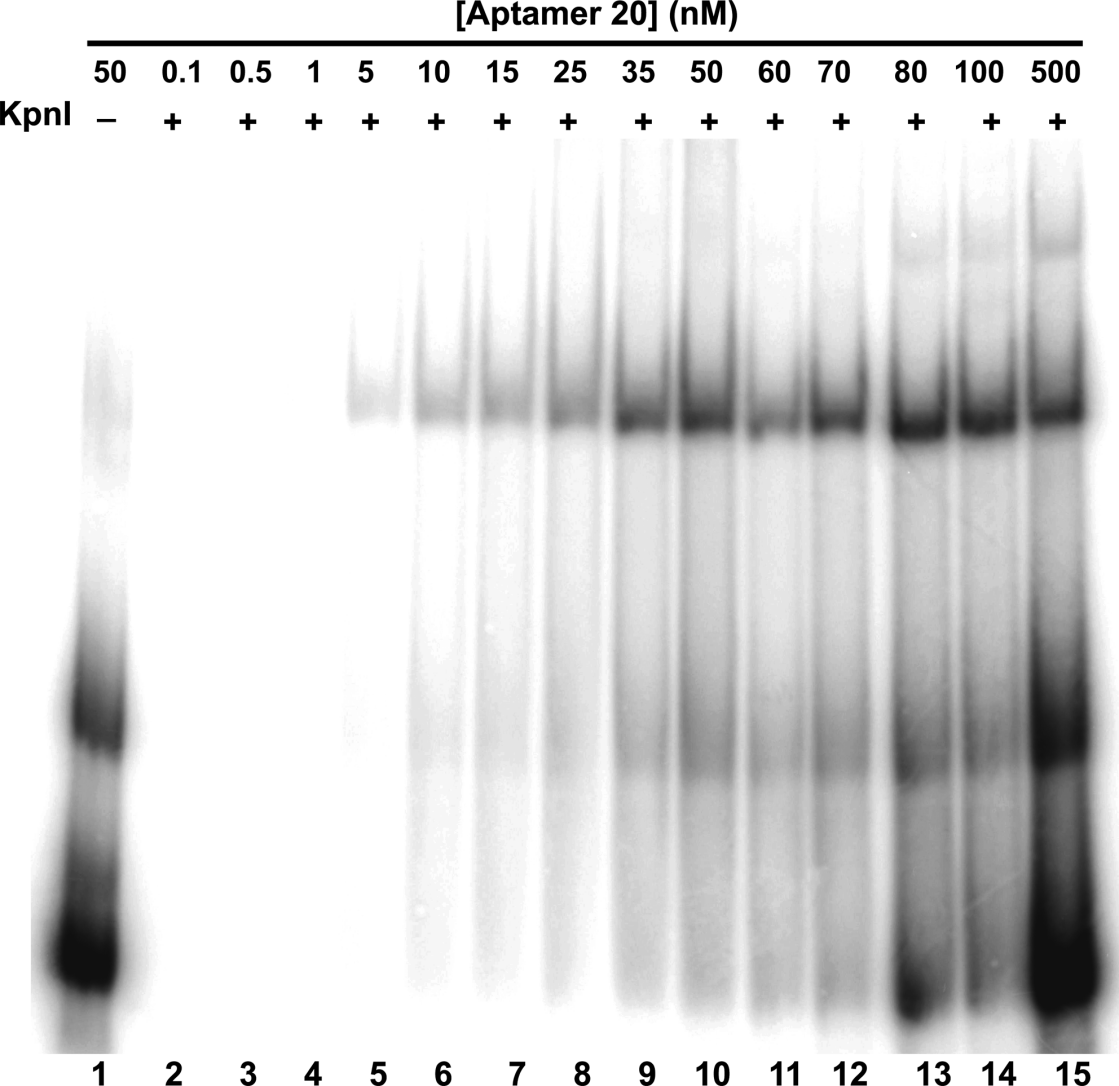
Titration of 50 nM KpnI with high affinity radiolabeled aptamer 20 in the concentration range 100 pM–500 nM.

Similar aptamers 20 and 30 displayed tight binding to KpnI, with *K*_d_ values of 11.6 ± 2.3 nM and 18.6 ± 4.5 nM, respectively (Figure 4A, D and Table 4). Aptamers 24 and 29 displayed *K*_d_ values ∼2- and ∼25-fold weaker specificity, respectively (Figure 4B and C). As shown in Table 4, fit values of the Hill coefficient were greater than unity, typically suggestive of positive cooperativity in multi-ligand binding reactions. The data in Figure 3, lanes 10–15 indeed showed some evidence of multiple KpnI proteins bound per aptamer at very high protein concentration, perhaps contributing to Hill coefficients. However, only a single (apparently 1:1) aptamer monomer/KpnI dimer complex was detected when a fixed concentration of KpnI protein (50 nM) was incubated with a wide range of aptamer concentrations (see example in Figure 5). There is only a small trace of higher molecular weight complex in the presence of high excess anti-KpnI aptamer (Figure 5, lanes 13–15). This suggests that single anti-KpnI RNA aptamers bind KpnI protein dimers.

### Quantitating KpnI inhibition by RNA aptamers

**Figure 6.**
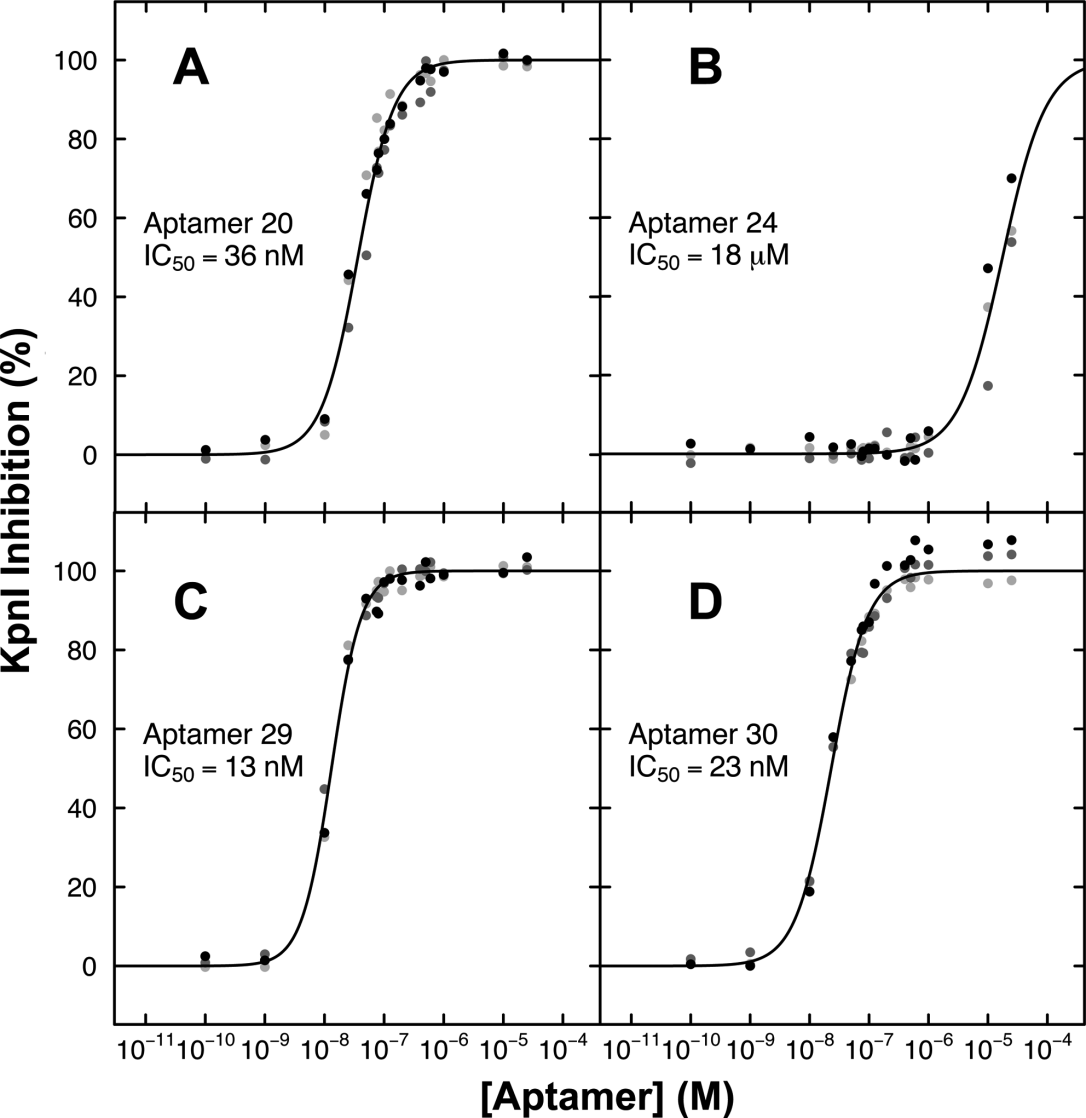
KpnI inhibition by anti-KpnI RNA aptamers. KpnI in the presence of 20 nM fluorescent DNA probe was incubated with anti-KpnI aptamers in the concentration range 10 pM–100 μM. (**A**) Aptamer 20; (**B**) aptamer 24; (**C**) aptamer 29; (**D**) aptamer 30.

Quantitation of enzyme inhibition was performed by titrating, in triplicate, 10 pM–100 μM final concentrations of 15 anti-KpnI aptamers in cleavage reactions containing 20 nM fluorescent DNA probe and 0.2 U/μl KpnI in standard KpnI digestion buffer. The aptamer concentration to inhibit 50% of probe cleavage under these conditions (IC_50_) was determined by curve-fitting and estimations of uncertainty by Monte Carlo simulations, see ‘Materials and Methods’ section. IC_50_ values ranged between 13 nM and 18 μM depending on the aptamer. Representative examples are shown in Figure 6 and Table 5. Similar anti-KpnI aptamers 20 and 30 were the most powerful selective inhibitors, with IC_50_ of values 36.1 ± 3.9 nM and 23.1 ± 0.8 nM, respectively, in this assay (Figure 6A and D). Interestingly, aptamer 24 shown in Figure 4B to have tight binding affinity, was a poor KpnI inhibitor (IC_50_ ∼ 18 ± 5.4 μM) (Figure 6B). Aptamer 29 was relatively weaker in both binding and inhibition (Figures 4C and 6C).

**Table 5.** Relative half maximal inhibitory concentration (IC_50_) and slope factor (*s*) for selected aptamers inhibiting KpnI

Aptamer^a^	*s*	IC_50_
20	1.45 ± 0.14	36.1 ± 3.9 nM
24	1.72 ± 2.09	18.0 ± 5.4 μM
29	1.92 ± 0.16	153 ± 11 nM
30	1.43 ± 0.07	23.1 ± 0.8 nM
20s	1.77 ± 0.25	13.0 ± 1.5 nM

^a^Parameters reported with uncertainty reflect the mean and standard deviation of 10 000 fits to experimental data (at least three replicates) with added Gaussian noise (see ‘Materials and Methods’ section).

**Figure 7.**
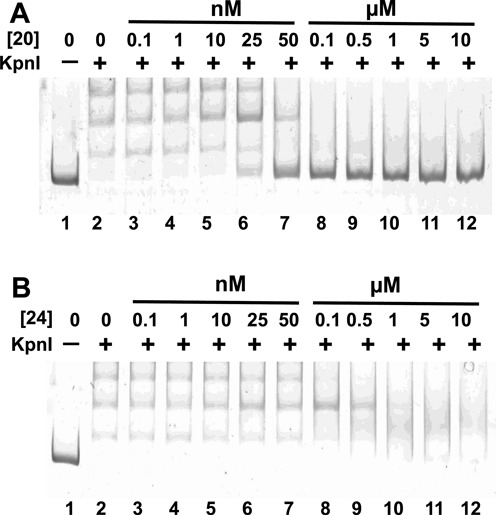
Competition gel shift assay showing inhibition of KpnI binding to fluorescent DNA probe in the presence of 100 pM–10 μM anti-KpnI RNA aptamers. (**A**) Aptamer 20, (**B**) aptamer 24.

We hypothesized that the selected anti-KpnI RNA aptamers function as competitive inhibitors of KpnI. This was tested by monitoring the ability of anti-KpnI RNA aptamers to compete with the fluorescent DNA probe for binding to KpnI protein. The results of two examples, aptamer 20 and 24 are shown in Figure 7. Under these binding conditions commercial KpnI preparations show multiple shifted DNA complexes due to partial protein oxidation (Figure 7A, lanes 2–6 and Figure 7B, lanes 2–12). Interestingly, aptamer 20 (strong binding and strong inhibition) acts as a competitor, as expected, (Figure 7A), while aptamer 24 (strong binding but weak inhibition) does not (Figure 7B). This suggests that aptamer 24 may bind KpnI without occluding its active site.

### Specificity of REase inhibition

We initially determined that even 40 μM anti-KpnI RNA aptamers did not inhibit DNA cleavage by BamHI or PacI (Table 2). We considered a more stringent test of specificity by challenging the KpnI isoschizomer Acc65I (identical DNA target sequence specificity) with aptamer 20. Remarkably, this aptamer is completely specific for KpnI (Supplementary Figure S1). This result suggests that the DNA sequence mimicry of anti-KpnI aptamer 20 must include an element of protein interaction outside the minimal DNA recognition interface.

### Anti-KpnI RNA aptamer secondary structures

**Figure 8.**
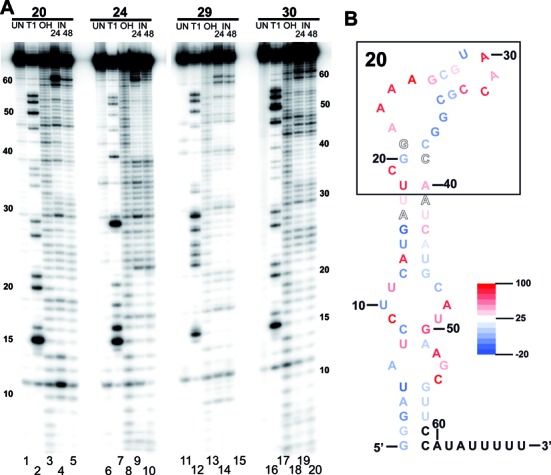
(**A**) In-line probing (36) of high-affinity anti-KpnI aptamers 20, 24, 29, 30. (UN: unmodified samples (lane 1, 6, 11 and 16); T1: RNAse T1 digestions (lane 2, 7, 12 and 17); OH: alkaline hydrolysis reactions (lane 3, 8, 13 and 18); IN: In-line attack reactions after 24 h (lane 4, 9, 14 and 19) and 48 h (lane 5, 10, 15 and 20). (**B**) Predicted secondary structure of aptamer 20 and validation by in-line probing shown as color-coded heat map (red, high in-line attack rate; white, intermediate in-line attack rate; blue, low in-line attack rate). Black box highlights the nucleotides that were randomized in the original library.

The RNA aptamer library employed in these selections used as a scaffold a compact hairpin inspired by the high-affinity anti-NF-κB p50 RNA aptamer (12,17,23). To test if this structural framework was conserved after selection, secondary structure predictions for anti-KpnI aptamers 20, 24, 29 and 30 were generated (Figure 8B and Supplementary Figure S2). Indeed, predicted lowest-energy folds for all aptamers retain the hairpin approximate, each with bulged and unpaired nucleotides derived from random regions and the invariant tetraloop and paired stem elements approximately as expected. We note that nucleotides predicted to be in unpaired internal loops often are discovered to be participating in complex non-canonical interactions (23).

The validity of these predicted secondary structures was tested by in-line probing analysis (27). This technique exploits the intrinsic chemical instability of RNA due to spontaneous nucleophilic attack of a 2′ hydroxyl oxygen on the adjacent phosphorous center. This spontaneous attack is accelerated when the nucleophile is ‘in-line’ with the adjacent phosphorus center and the 5′ oxygen-leaving group of the linkage, a scenario that is suppressed in conventional Watson–Crick base-paired stems (27–29,36). In-line probing reactions for anti-KpnI aptamers 20, 24, 29 and 30 are shown in Figure 8A. We compared uniform alkali cleavage (Figure 8A, lanes 3, 8, 13, 18) to in-line attack lanes (Figure 8A, lanes 4/5, 9/10, 14/15, 19/20, respectively) and a heat map was generated showing low to high in-line attack rates at base pair resolution. An example for aptamer 20 is shown in Figure 8B and the remaining cases in Supplementary Figure S2. As shown in Figure 8B, nucleotides predicted to be base-paired tend to show suppressed in-line attack rate, as expected (Figure 8B, G1–A6, G34–C39, A41–C47 and G55–U58) corresponding to heat-map scores in shades of blue. Regions of flexibility or structures where the 2′ OH group more frequently samples an in-line conformation have higher rates of self-cleavage, as predicted (Figure 8B, A22–G26, A30–C33, A48–G50 and A52–C54) and shown in shades of red in heat map (Figure 8B and Supplementary Figure S2). We note that the RNA secondary structure prediction algorithm used here does not take into account the relative stability of A–G mispairs (30). There are potential pairs of this kind (for example A25–G36 and A6–G53) and such pairs might help explain differential in-line attack profiles on opposite strands of a potential double helix.

Based on the secondary structure prediction and in-line probing data for anti-KpnI aptamer 20 (Figure 8B), we tested if a minimal stem-loop structure from positions 12–46 (aptamer 20s) was sufficient for KpnI inhibition (Table 3). Indeed, this 38-nt RNA aptamer is a potent KpnI inhibitor (Supplementary Figure S3).

### Further study of minimal recognition sequence

To further explore minimal recognition sequences of the anti-KpnI aptamers we created four mutants of aptamer 20. A scrambled version of aptamer 20 was created (20_scram) in which the nucleotide sequences of the random regions (N10 and N9) were randomized. A 26-nt truncated variant of aptamer 20 (20_26 nt) presented a shorter version of the putative recognition hairpin. A variant of aptamer 20 (20_AAA) was mutated in a distinctive stretch of four adenosine residues. Finally, a variant aptamer (20_downst) contains mutations within the N9 random region downstream of the tetraloop (Table 3). Secondary structure predictions for these variant aptamers suggest that only 20_AAA and 20_downs conserve aspects of aptamer 20 structure, while 20_26 nt and 20_scram differ greatly (Supplementary Figure S4). Importantly, digestion inhibition experiments showed that all four of these mutant aptamers lost inhibitory activity (IC_50_ values in the hundreds of micromolar; Supplementary Figure S5.) These results confirm that KpnI binding involves nucleotides mapping to the random region of the stem-loop, as expected.

### CD spectroscopy

In an attempt to evaluate the hypothesis that restriction inhibition involves DNA mimicry by nucleotides within the stem-loop domain of aptamer 20, we compared CD spectra of aptamers 20, 20s, 20_scramble, 20s_scramble, 20_26 nt, 20_AAA, 20_downs, with known DNA mimic anti-NF-κB p50 serving as control. As shown in Supplementary Figures S6 and S7, CD spectra for all of these molecules are dominated by the signal of the overall RNA stem-loop structure [265 nm (+), 240 nm (−), 220 nm (+) and 210 nm (−) (31–33)]. In addition, difference spectra were not interpretable. This result indicates that CD spectroscopy is not well-suited to drawing conclusions about potential B-DNA mimicry within a small sub-domain of an RNA aptamer.

## DISCUSSION

Here we report the *in* *vitro* selection and characterization of RNA aptamers using REases BamHI, KpnI and PacI as targets. We identify RNA aptamers selected against KpnI that selectively bind (*K*_d_ ∼12–30 nM) and inhibit (IC_50_ ∼20–150 nM) this REase with high specificity. Evaluation of four representative anti-KpnI aptamers (20, 24, 29 and 30) by reverse titration and by competition studies suggests a 1 aptamer:1 KpnI homodimer binding stoichiometry. Binding appears to be at or near the KpnI DNA binding site, competing with DNA binding. Binding selectivity can be exquisite: mimicry of the KpnI DNA binding site by aptamer 20 does not confer inhibition of KpnI isoschizomer Acc65I. This suggests that aptamer 20 contacts KpnI surfaces beyond the minimal DNA recognition interface.

Secondary structure prediction, in-line probing experiments and CD spectra suggest that the selected aptamers evolved on the approximate intended hairpin. KpnI inhibition by a minimal version of aptamer 20 (aptamer 20s) confirms this prediction. Interestingly, none of the inhibitory anti-KpnI aptamers carries any obvious homology to the duplex DNA binding site 5′-GGTACC. This is not unexpected in that the previously-studied high-affinity anti-NF-κB p50 RNA aptamer also does not bear obvious primary sequence homology to the DNA sequence it mimics (12,17,23).

From the study of several aptamer 20 mutants we deduce that nucleotides C12–U17 of the sequence 5′-CAUGAU are crucial for promoting the suggested hairpin structure necessary for target binding. Disruption of this sequence inactivates restriction inhibition (Supplementary Figures S3 and S5). We also found that disruption of either the distinctive A_4_ sequence within the N10 domain or the N9 sequence downstream from the tetraloop inactivated function even when an overall hairpin structure was roughly conserved (Figure 8B and Supplementary Figure S4). This confirms that the minimal structure within the 31-nt 20s depends on the selected random region, as expected. Future studies could vary the character of the tetraloop, a feature found important in the case of the anti-NF-κB RNA aptamer, where loop optimization afforded a ∼20-fold improvement in activity (16).

Regrettably, there are no high-resolution structural data for KpnI, a member of the HNH superfamily. Homology analysis suggests that amino acid residues D148, H149 and Q175 compose the catalytic metal-binding active site of KpnI (34). Furthermore, CD spectra of aptamer 20 and several aptamer 20 variants were substantially similar and comparable to that of the anti-NF-κB RNA aptamer structure that had been the basis for the design scaffold Supplementary Figures S6 and S7). This result confirms that the anti-KpnI aptamers evolved within the intended hairpin scaffold and this structure dominates the CD spectrum, preventing conclusions to be drawn about details of B-DNA mimicry within a small region of the structure. We predict that eventual high-resolution structure determination for complexes of KpnI and anti-KpnI RNA aptamers will reveal RNA binding that obscures this active site. Based on our previous studies of the anti-NF-κB RNA aptamer we hypothesize that the anti-KpnI aptamers reported here have evolved as structural mimics of the DNA major groove recognized by KpnI.

Our prior selections of RNA aptamers against NF-κB family members (17,35) suggest that DNA mimics may be the primary products for DNA binding protein targets. The anti-NF-κB p50 aptamer studied in greatest detail was characterized by a predicted hairpin structure with asymmetric internal loop. There was no obvious sequence similarity to NF-κB binding sites in DNA and no hint from chemical or enzymatic probing as to the mechanism of protein recognition. Only with the solution of X-ray co-crystals of the aptamer and its protein target and the determination of the aptamer solution structure by nuclear magnetic resonance did it become clear that the aptamer had been selected for elegant structural mimicry of the DNA major groove, allowing the RNA to engage precisely the same face of the transcription factor that normally engages DNA. We therefore hypothesize that RNA aptamers selected against DNA binding proteins are likely to evolve interfaces based on DNA mimicry. It remains to be seen through high-resolution structural studies if that is the mechanism of the anti-KpnI RNA aptamers described here. By designing the present selections in the context of an RNA structural scaffold based on the previously-studied anti-NF-κB RNA aptamer, our ultimate goal is to test the hypothesis that DNA groove mimicry is a general mechanism for RNA binding to DNA binding proteins. In this sense, the new anti-KpnI RNA aptamers described here are now candidates for high-resolution structural study to judge the validity of this hypothesis.

It is intriguing that RNA aptamers selected against BamHI and PacI showed binding activity without enzyme inhibition. We hypothesize that three considerations are at play. First, these enzymes may have cationic surfaces that elicit RNA ligands lacking the ability to inhibit enzyme activity. Second, we found it necessary to identify different stringent salt conditions for selections with each enzyme. This means that the selection conditions for BamHI (very high salt in the presence of Mg^2+)^ and for PacI (very low salt in the presence of Ca^2+^) are least like the actual conditions for enzyme activity. Finally, we note that BamHI selections employed catalytically inactive E111A mutant protein. It is possible that the selected aptamers do not bind as well to catalytically-active wild-type protein.

When KpnI inhibitory RNA aptamers were discovered, we considered the possibility that DNA mimicry might be so effective that RNA cleavage by KpnI could result. Such RNA cleavage was not observed among the studied aptamers (data not shown). However, it is possible that RNA cleavage might rapidly inactivate such aptamers so they do not function as competitive inhibitors.

To our knowledge, the anti-KpnI RNA aptamers reported here are the first known selective restriction enzyme inhibitors. Current approaches to REase inhibition are limited to irreversible heat denaturation or Mg^2+^ chelation. Neither approach is selective, reversible or applicable *in* *vivo*. Anti-REase RNA aptamers could prove valuable in studies of restriction enzyme mechanism. Moreover, these aptamers provide new examples in the growing list of natural and artificial RNAs that bind DNA-binding proteins by DNA mimicry. As such, they provide the raw material for future structural studies to understand in detail this DNA mimicry and to deduce principles by which an RNA scaffold might be designed to inhibit transcription factors with known DNA binding specificities.

## Supplementary Material

SUPPLEMENTARY DATA
