# Supplementary material for: RNA aptamer inhibitors of a restriction endonuclease
Source: Nucleic Acids Res. 2015 Jul 15;43(15):7544–55. doi: 10.1093/nar/gkv702 (PMC4551934; doi:10.1093/nar/gkv702)
Supplement: SUPPLEMENTARY DATA [file supp_43_15_7544__index.html]

RNA aptamer inhibitors of a restriction endonuclease — RNA aptamer inhibitors of a restriction endonuclease — SUPPLEMENTARY DATA 

# RNA aptamer inhibitors of a restriction endonuclease

## SUPPLEMENTARY DATA

- SUPPLEMENTARY DATA
