# Supplementary material for: RNA aptamer inhibitors of a restriction endonuclease
Source: Nucleic Acids Res. 2015 Jul 15;43(15):7544–55. doi: 10.1093/nar/gkv702 (PMC4551934; doi:10.1093/nar/gkv702)
Supplement: SUPPLEMENTARY DATA [file supp_gkv702_Mondragon_and_Maher_supplemental.pdf]

Supplementary data for Mondragon and Maher
RNA aptamer inhibitors of a restriction endonuclease

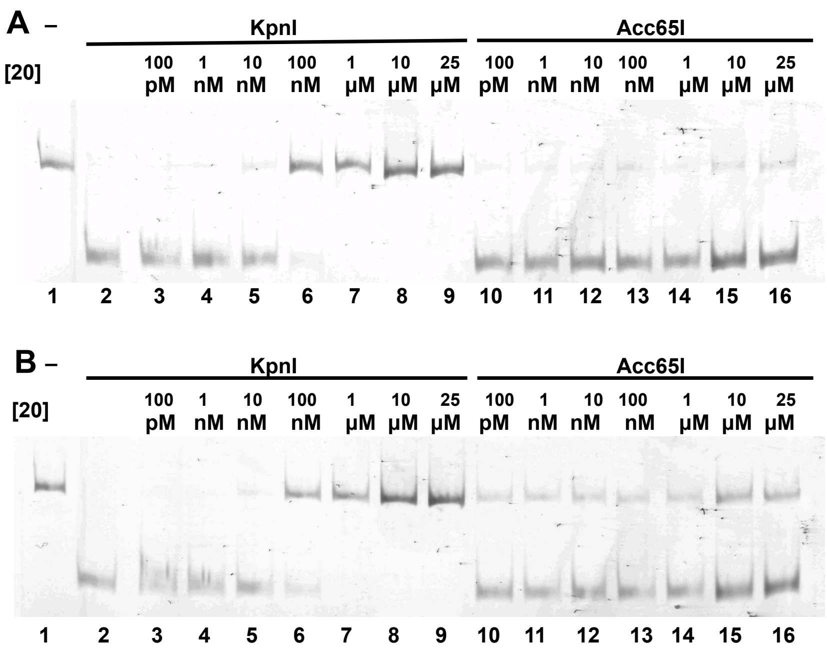

Supplemental Figure S1. Effects of anti-KpnI RNA Aptamer 20 on KpnI isoschizomer Acc65I. A. Comparison of KpnI and Acc65I digestion inhibition of 20 nM fluorescent DNA probe in the presence of the indicated concentrations of Aptamer 20, each enzyme in the buffer recommended by the manufacturer (NEB). B. Comparison of digestion inhibition of 20 nM fluorescent DNA probe by either KpnI or Acc65I in NEB buffer 2.1 (50 mM NaCl, 10 mM Tris-HCl, 10 mM $MgCl_2$, 100 μg/mL BSA, pH 7.9 at 25 °C) in which both enzymes show 75% activity.

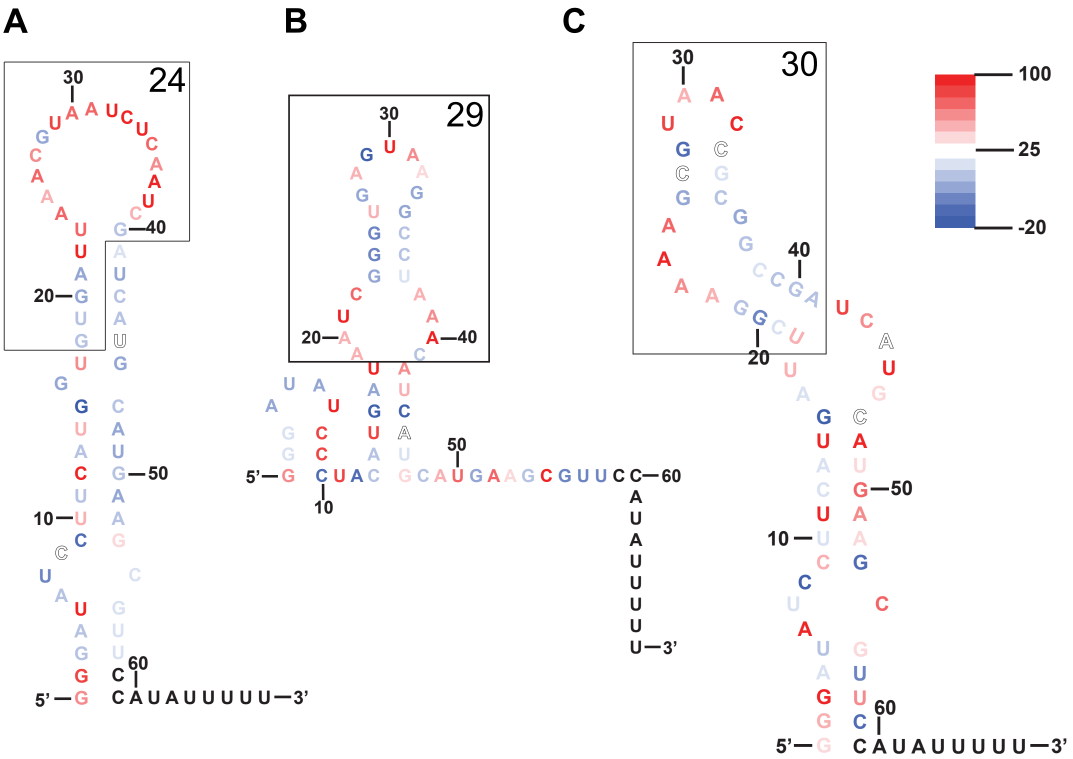

Supplemental Figure S2. Predicted secondary structures of high affinity anti-KpnI aptamers, color-coded based on heat map generated by analysis of In-line attack assay from Figure 8.

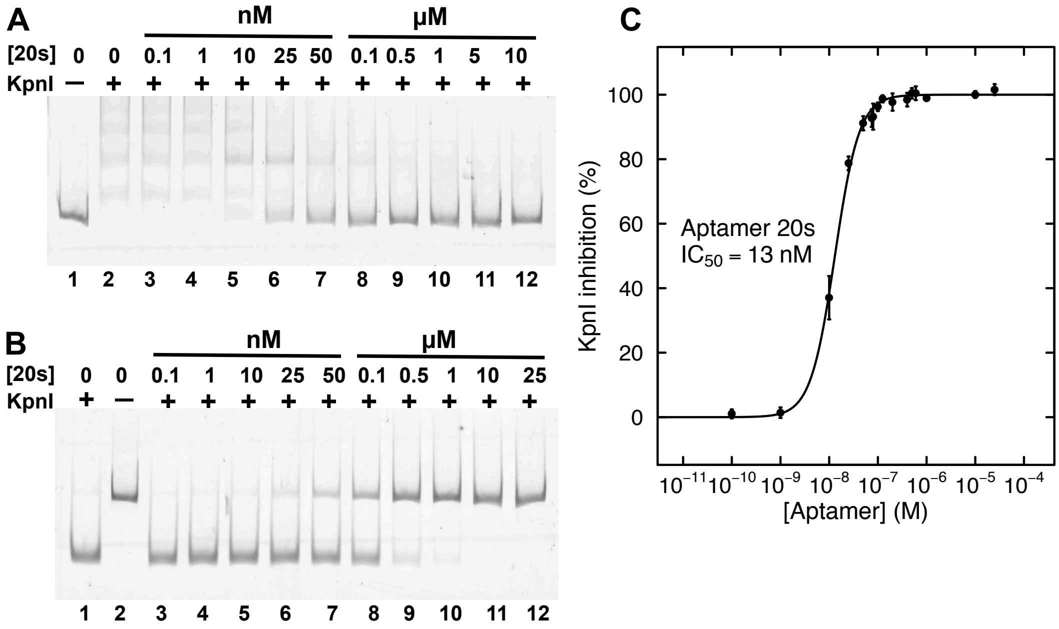

Supplemental Figure S3. A. Competition gel shift assay showing inhibition of KpnI binding to 20 nM fluorescent DNA probe in the presence of 100 pM - 10 µM concentrations of 38-nucleotide derivative of RNA Aptamer 20 (Aptamer 20s). B. KpnI inhibition by Aptamer 20s. C) Quantitation of panel B, digestion inhibition of KpnI when titrated with 100 pM to 10 µM of Aptamer 20s.

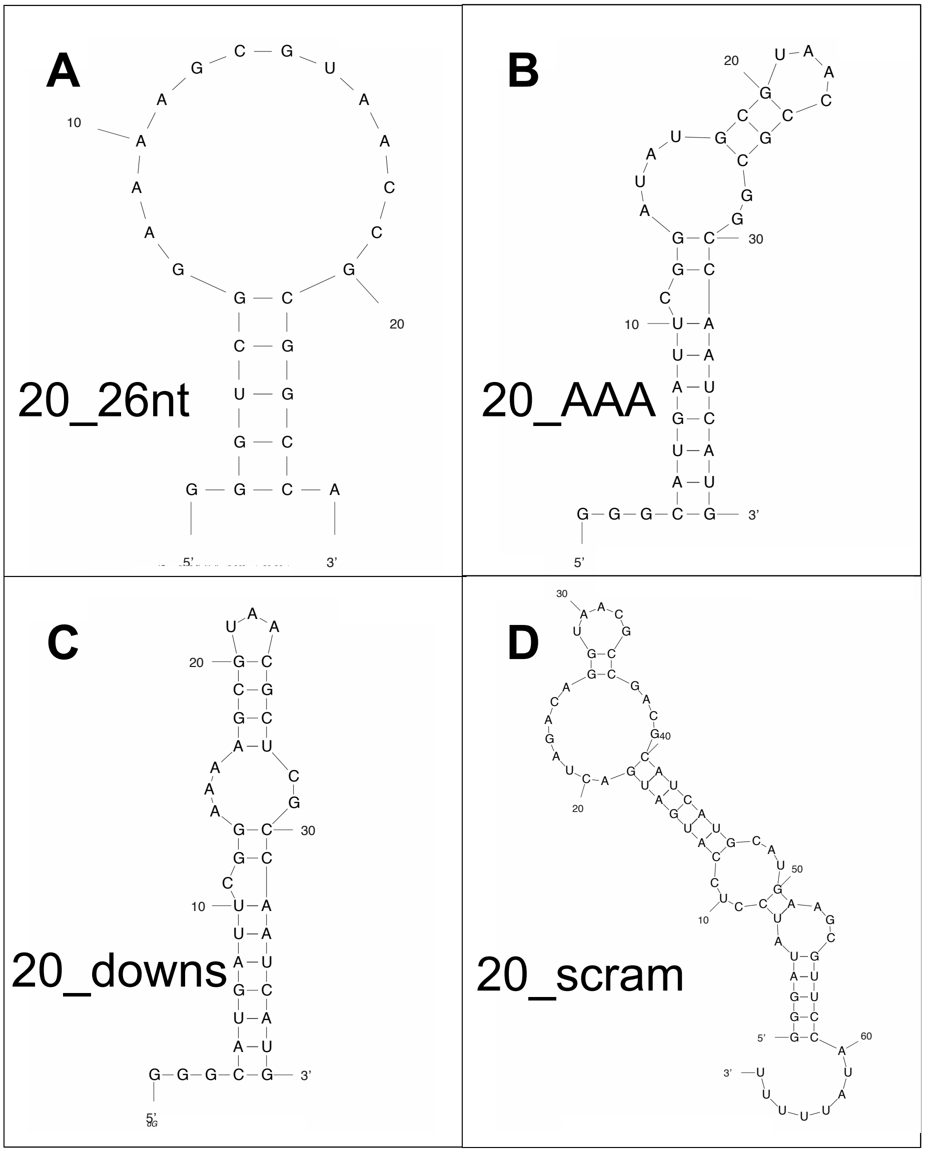

Supplemental Figure S4. Predicted secondary structures of the indicated mutant versions of Aptamer 20s.

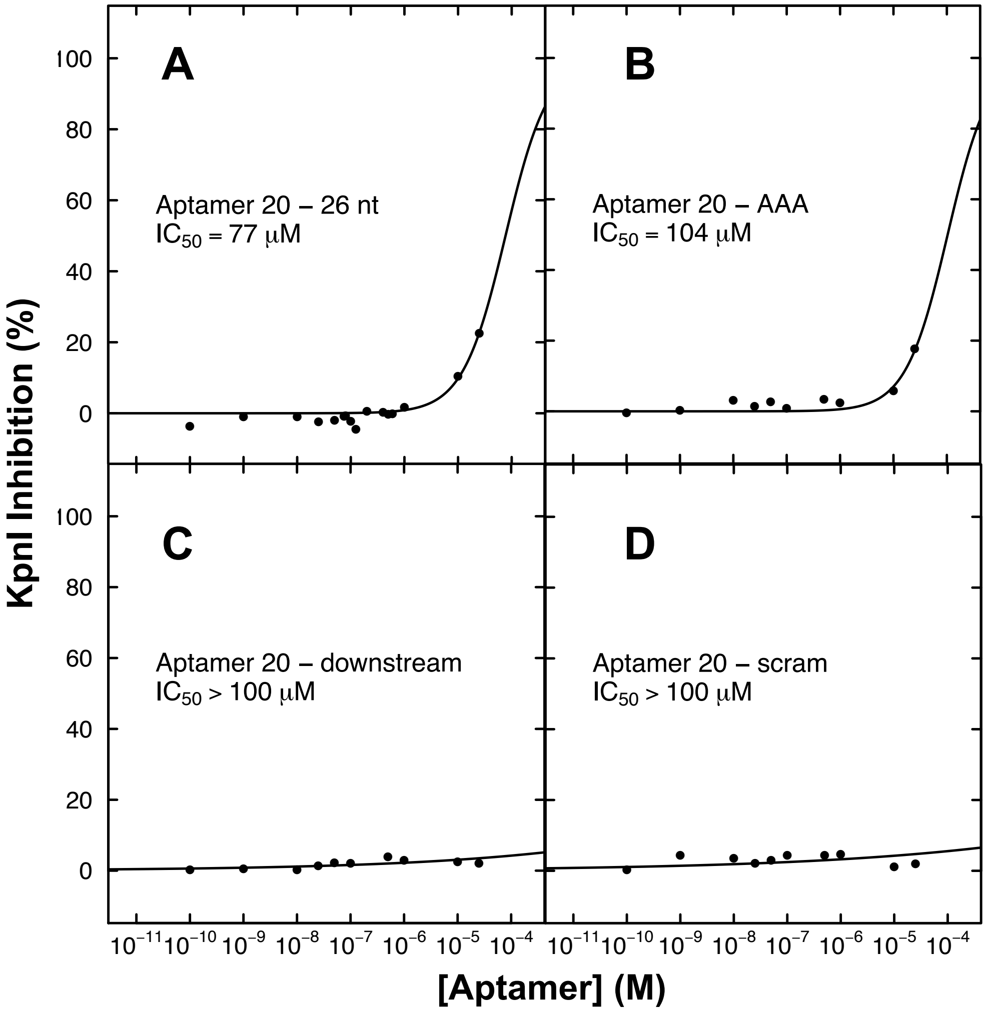

Supplemental Figure S5. KpnI enzyme inhibition data and derived parameters for Aptamer 20s mutants.

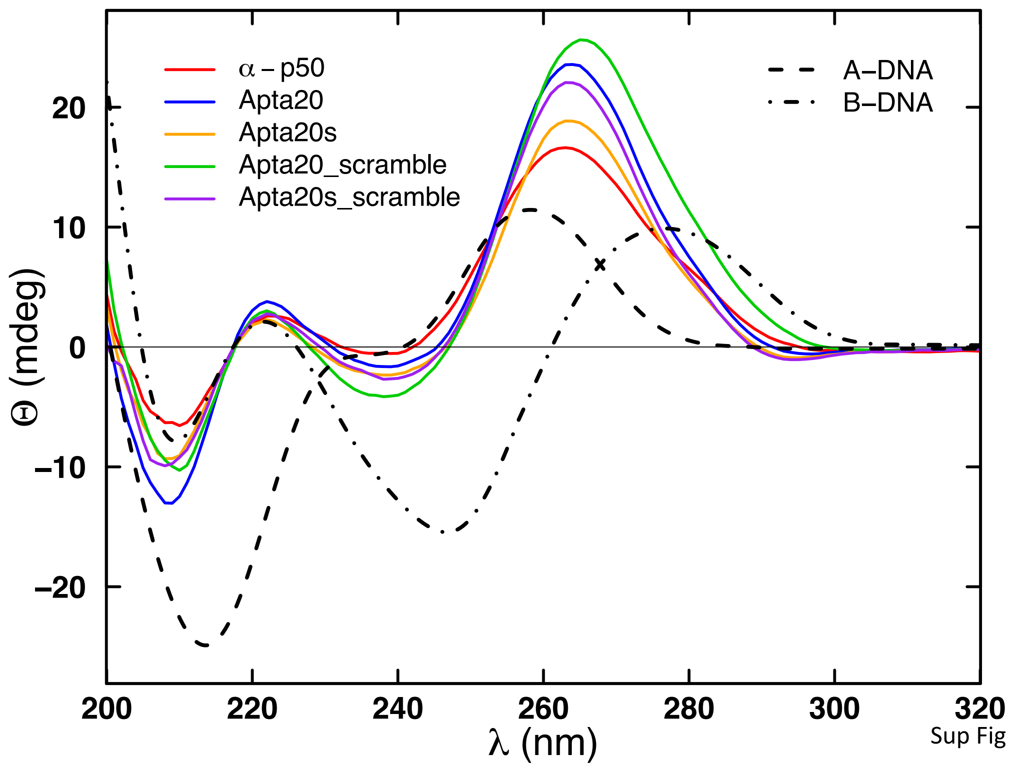

Sup Fig S6

Supplemental Figure S6. Experimental CD spectra of the indicated RNA aptamers compared to canonical A- and B-form DNA. Aptamer CD spectra are dominated by the overall hairpin conformation of the RNA, preventing interpretation of evidence for B-like substructure.

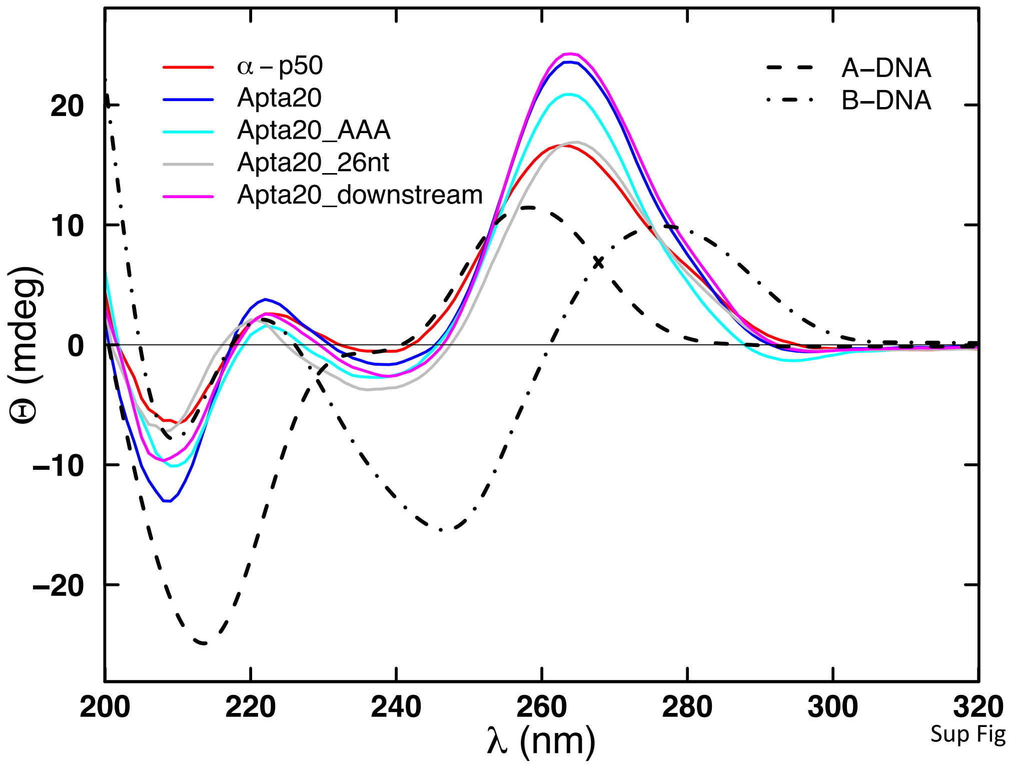

Sup Fig S7

Supplemental Figure S7. Experimental CD spectra of the indicated RNA aptamers compared to canonical A- and B-form DNA. Aptamer CD spectra are again dominated by the overall hairpin conformation of the RNA, preventing interpretation of evidence for B-like substructure.
